# Emulating Clinical Trials with the Mayo Clinic Platform: Cardiovascular Research Perspective

**DOI:** 10.1101/2025.03.19.25324271

**Published:** 2025-03-24

**Authors:** Xiaodi Li, Sivaraman Rajaganapathy, Xinyue Hu, Jingna Feng, Jianfu Li, Yue Yu, Phil Fiero, Soulmaz Boroumand, Richard Larsen, Xiaoke Liu, Cui Tao, Nansu Zong

**Affiliations:** 1 Department of Artificial Intelligence and Informatics, Mayo Clinic, Rochester, MN, USA; 2 MCHS Cardiology, Mayo Clinic, Rochester, MN, USA; 3 Mayo Clinic Platform, Mayo Clinic, Rochester, MN, USA

**Keywords:** RCT emulation, real-world data, electronic health records, cardiovascular treatment, observational study

## Abstract

**Background:**

Randomized controlled trials (RCTs) provide the highest level of clinical evidence but are often limited by cost, time, and ethical constraints. Emulating RCTs using real-world data (RWD) offers a complementary approach to evaluate the treatment effect in a real clinical setting. This study aims to replicate clinical trials based on Mayo Clinic Platform (MCP) electronic health records (EHRs) and emulation frameworks. In this study, we address two key questions: (1) whether clinical trials can be feasibly replicated using the MCP, and (2) whether trial emulation produces consistent conclusions based on real clinical data compared to the original randomized controlled trials RCTs.

**Methods:**

We conducted a retrospective observational study with an adaption of trial emulation. To assess feasibility, we applied a refined filtering method to identify trials suitable for emulation. The emulation protocol was carefully designed on top of the original RCT protocol to balance scientific rigor and practical feasibility. To minimize potential selection bias and enhance comparability between groups, we employed propensity score matching (PSM) as a statistical adjustment method.

**Results:**

Based on our predefined search criteria targeting phase 3 trials focused on drug repurposing for heart failure patients, we initially identified 27 eligible trials. After a two-step manual review of the original eligibility criteria and extraction of the patient cohorts based on MCP visualizer, we further narrowed our selection to the WARCEF trial, as it provided an adequate sample size for the emulation within the MCP. The experiment compares the WARCEF trial and a simulation study on Aspirin vs. Warfarin. The original study (smaller sample) found no significant difference (HR = 1.016, p < 0.91). The simulation (larger sample) showed a slightly higher HR (1.161) with borderline significance (p < 0.052, CI: 0.999–1.350), suggesting a possible increased risk with Warfarin, though not conclusive.

**Conclusion:**

RCT emulation enhances real-world evidence (RWE) for clinical decision-making but faces limitations from confounding, missing data, and cohort biases. Future research should explore machine learning-driven patient matching and scalable RCT emulation. This study supports the integration of RWE into evidence-based medicine.

## 1. Introduction

Randomized controlled trials (RCTs) are widely regarded as the gold standard for establishing causal relationships in clinical research due to their ability to minimize bias and confounding [1]. However, RCTs are often expensive, time-consuming, and ethically or logistically infeasible for certain research questions [2]. In response to these limitations, researchers are increasingly using observational data and real-world evidence (RWE) to complement RCT findings and support clinical decision-making [3, 4]. By using structured methodologies, researchers can design observational studies to assess real clinical outcomes of drugs, providing comparisons to the reported results from clinical trials and offering a clearer picture of treatment effectiveness in real-world settings.

Many clinical decisions rely on observational data, particularly when RCTs are unavailable or impractical. However, observational studies often suffer from selection bias, immortal time bias, and confounding, which can distort causal inference. To mitigate these challenges, rigorous study design principles must be applied to ensure that observational analyses align as closely as possible with the conditions of a randomized trial. This requires a careful definition of eligibility criteria, treatment assignment methods, follow-up periods, outcome measures, and statistical analysis strategies to maintain the internal validity of the study [5, 6].

Since treatment assignment in observational studies is not randomized, methods such as propensity score matching (PSM), inverse probability of treatment weighting (IPTW), and instrumental variable analysis are employed to adjust for potential biases and improve the validity of causal inferences [7, 8]. These methods help address common biases, including immortal time bias and confounding by indication [9, 10]. In addition to controlling for bias, clearly defined outcome measures and robust statistical modeling further strengthen the reliability of observational findings [11, 12]. However, the primary challenge may not lie in the statistical methods themselves but rather in data processing and the adaptation of the original protocol to ensure both scientific rigor and feasibility in a real-world clinical setting, such as within MCP. Efficient data harmonization, replication of study design, and integration of heterogeneous real-world data sources are critical to maintaining the integrity of causal inferences while ensuring practical implementation in clinical environments. The main development focused on translating raw data from MCP, filtering it, and navigating the database to accurately identify, extract, and process the necessary information.

Observational research has been successfully applied in numerous medical fields, including cardiovascular research, diabetes treatment evaluation, cancer drug development, and COVID-19 treatment studies [13]. When properly designed and analyzed, observational studies can yield findings that closely align with RCT results, reinforcing the credibility of real-world evidence in clinical research. Large-scale initiatives such as the RCT-DUPLICATE project have demonstrated strong concordance between observational analyses and RCT outcomes, providing further validation of these methodologies as a complement to traditional trials [14]. Despite these advancements, challenges remain in the use of observational data for emulating RCTs. Issues such as missing data, unmeasured confounders, and cohort selection biases can impact study outcomes, particularly when working with electronic health records (EHRs) [15, 16]. While RCTs remain the cornerstone of evidence-based medicine, integrating real-world data enhances nonrandomized studies, bridging the gap to improve patient care and decision-making [17, 18].

This study aims to emulate randomized clinical trials (RCTs) using observational data from the Mayo Clinic Platform (MCP) to evaluate the comparative effectiveness of different medicines in patients. By leveraging electronic health records (EHRs) and real-world data (RWD), the study seeks to replicate the design and analytical rigor of RCTs, ensuring that findings align with traditional trial outcomes. A key focus is on constructing well-defined patient cohorts that closely mirror those in RCTs. Using schema visualizers and workspaces, the study refines eligibility criteria, treatment assignments, follow-up periods, and outcome measures. Advanced statistical methods such as propensity score matching (PSM) is applied to minimize confounding and approximate randomization. The primary outcome is a composite endpoint of ischemic stroke, intracerebral hemorrhage, or death, with additional analyses extending to other heart failure drug repurposing trials, including WARCEF, RAID, HEpEF, GIPS III, and RELAX. This research also aims to address methodological challenges such as missing data, confounding variables, and cohort selection difficulties. The study validates emulation results against existing RCT findings to ensure accuracy and reliability. Ultimately, this work contributes to the integration of real-world evidence into clinical and regulatory decision-making, supporting a more efficient and cost-effective approach to trial emulation.

Our method makes a threefold contribution: (1) demonstrating the feasibility of RCT emulation using real-world data (RWD) within MCP, ensuring that replication methods can be systematically applied in clinical settings; (2) addressing methodological challenges in observational research, such as data processing complexities, confounder balancing, and adapting trial protocols for structured and reproducible replication; (3) providing an approach to systematically evaluate RCT replication using MCP, assessing whether findings from real-world data can consistently reproduce original trial results under controlled conditions.

## 2. Methods

The study is conducted in compliance with institutional review board (IRB) guidelines and data privacy regulations. De-identified patient data from MCP are used to protect participant confidentiality. This methodological approach aims to assess the feasibility of replicating RCT findings using real-world data (RWD) within MCP, ensuring methodological rigor and comparability to original trial conclusions, providing valuable insights into the effectiveness of Warfarin and Aspirin in heart failure patients while advancing the use of RWD in clinical research. We divide our method as two components: (1) filtering, and (2) emulation.

### 2.1. Filtering

**Figure 1.**
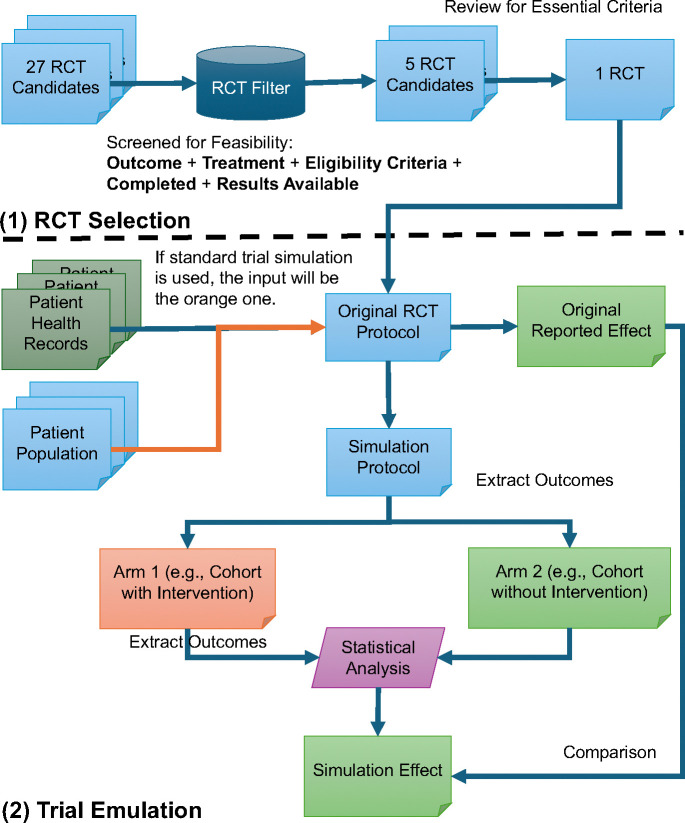
Overview of the proposed method. The figure illustrates the study’s RCT emulation process using real-world data (RWD) from the Mayo Clinic Platform (MCP). It consists of three main components: (1) RCT selection, where RCT candidates are filtered based on essential criteria (outcome, treatment, eligibility criteria, completed, and results available); (2) trial emulation process, where patient data from electronic health records (EHRs) are used to form intervention and non-intervention cohorts for outcome comparison.

As, shown in Figure 1, part (1), we are provided with 27 RCTs Select Randomized Clinical Trial (RCT) Candidates that for Heart Failure Drug Repurposing, Phase 3 Completed trials. We selected five trials from the 27 candidates for convenience to determine the final trial. After evaluating the complexity and feasibility of each trial, we ultimately chose the WARCEF trial for our emulation. To streamline the selection process, we applied filtering criteria including outcome, treatment, eligibility criteria, completed, and results available, and narrowed the pool to five trials for further evaluation. This study is a retrospective observational analysis designed to emulate randomized controlled trials (RCTs) using real-world data (RWD) from the Mayo Clinic Platform (MCP), as shown in Figure 1, part (2). Compared to the standard trial simulation framework as shown in Figure 1, part (3), the study utilizes electronic health records (EHRs) and other structured healthcare data to compare the effectiveness of Warfarin versus Aspirin in patients with reduced cardiac ejection fraction. The study follows a structured approach to RCT emulation, ensuring that the design, patient selection, treatment assignment, and statistical analyses align with traditional interventional trials.

The application of multiple participation criteria inevitably reduces cohort size, which can, in turn, diminish statistical power. Existing literature suggests that many studies use only a subset of participation criteria when building cohorts, highlighting the need to carefully balance cohort selection and statistical validity. To minimize bias while ensuring the identification of the most relevant RCT criteria, we categorize selection criteria into three groups: Essential Criteria, Confounding Variable Criteria, and Non-Essential Criteria.

Essential Criteria refer to factors directly related to the primary study variables and outcomes. These criteria define the key characteristics of the study population and ensure that selected patients meet the necessary conditions for meaningful comparisons. Confounding Variable Criteria address factors that, while not the primary focus of the study, are associated with both the treatment and the outcome, potentially influencing results if not properly adjusted. Lastly, Non-Essential Criteria represent conditions that are assumed to have already been applied in retrospective observational data. These criteria often include exclusions based on safety concerns, such as removing patients at high risk for adverse events. By systematically applying these criteria, we aim to refine cohort selection while preserving statistical power and ensuring robust comparisons in RCT emulation.

To enhance feasibility and maintain the integrity of our analysis, we adopted an Intention-to-Treat (ITT) [19] approach instead of a Per-Protocol (PP) [20] analysis. ITT ensures that all participants remain in their originally assigned treatment groups, regardless of adherence, preserving randomization benefits and reducing selection bias. This approach provides a more realistic assessment of treatment effectiveness in real-world settings, making it particularly suitable for our study objectives. In contrast, PP analysis, which includes only participants who strictly followed the protocol, could introduce bias by excluding non-adherent individuals, potentially limiting the generalizability of our findings.

### 2.2. Emulation

#### 2.2.1. Patient Cohort Selection

Patient cohorts are identified and refined using MCP’s Schema Visualizer and Workspaces to ensure comparability with traditional RCT populations. A base cohort was created using essential eligibility criteria, followed by a refined cohort with additional post-processing adjustments to improve comparability between treatment groups. The study applies predefined inclusion and exclusion criteria, including factors such as baseline cardiac function, comorbidities, prior anticoagulant use, and medication history.

We design a process to define time zero, determining when patients should be included. This process is based on two essential criteria. At time 0, both Criterion 1 and Criterion 2 are out of their respective selection regions. We set time zero 𝑡_!_ as the first time point when both Criterion 1 and Criterion 2 simultaneously enter their corresponding selection regions.

#### 2.2.2. Treatment Assignment and Outcome Measurement

For the WARCEF trial, patients are categorized into two groups: those receiving Warfarin and those receiving Aspirin. Since treatment assignment is not randomized in observational studies, statistical methods are employed to approximate randomization. Propensity score matching (PSM) and instrumental variable analysis are used to balance baseline characteristics and adjust for potential confounders.

The primary outcome of interest is a composite endpoint of ischemic stroke, intracerebral hemorrhage, or death. In our case, the death will be the most important outcome. Secondary outcomes include all-cause mortality, hospitalization rates, and adverse cardiovascular events. Outcomes are extracted from EHR-based clinical endpoints, ensuring alignment with previously published RCTs.

#### 2.2.3. Statistical Analysis

To compare treatment effects, Cox proportional hazards models are used to estimate time-to-event outcomes. Multivariable regression models will adjust for confounders, and sensitivity analyses will be conducted to assess the robustness of results. For subgroup analyses, stratified models will be developed based on age, sex, baseline ejection fraction, and comorbidities. Additionally, machine learning-based risk prediction models may be explored to refine patient matching and improve trial scalability in the future.

The Cox Proportional Hazards Model, introduced by Cox (1972) [21], is a widely used statistical method in survival analysis. It helps analyse the relationship between multiple factors and the likelihood of an event occurring over time, such as disease progression or treatment effectiveness. Unlike traditional survival models, the Cox model does not assume a specific pattern for survival times, making it flexible for various applications. It estimates the impact of different variables (such as age, gender, or lifestyle factors) on the risk of an event happening while allowing researchers to compare groups effectively. A key assumption of the model is that the effects of these variables remain consistent over time. Due to its ability to handle complex clinical and epidemiological data, the Cox model is extensively used in medical research, particularly in clinical trials and observational studies.

## 3. Results

### 3.1. Selected Studies

The summary and ranking of all trials are presented in Table 1, where WARCEF is ranked first. To identify the most suitable randomized controlled trial (RCT) for emulation, we conducted a structured selection process. We began by screening for completed trials with available results as of October 2024, identifying eight candidates. To ensure feasibility, we prioritized trials with outcome measures requiring only a single event-based assessment, such as mortality, cardiac events, or stroke, selecting the top five trials. For these candidates, we identified essential criteria, confounding variables, and non-essential criteria to guide cohort construction. Using the Cohort Visualizer tool, we then built patient cohorts based on essential criteria. The five RCTs were ranked according to three factors: how well the study population reflects real-world patients (favoring broader applicability), the number of essential criteria (with fewer being preferable), and the presence of a placebo cohort (preferring trials without one). Based on this evaluation, the WARCEF and RAID studies emerged as the top two candidates. Due to time constraints and our goal of testing the emulation capabilities of the MCP platform, we ultimately selected the WARCEF study, as it was the highest-ranked trial without a placebo cohort.

Following is the overview of each trial: (1) WARCEF: the title of this trial is Warfarin Versus Aspirin in Reduced Cardiac Ejection Fraction Trial. The primary outcome is Event Rate - Composite Endpoint of Ischemic Stroke, Intracerebral Hemorrhage, or Death. The intervention is Aspirin vs Warfarin. (2) RAID: the title of this trial is Ranolazine Implantable Cardioverter-Defibrillator Trial (RAID). The intervention is Ranolazine vs Placebo. (3) HEpEF: the title of this trial is Sildenafil in HFpEF (Heart Failure with Preserved Ejection Fraction) and PH. The intervention is Sildenafil vs Placebo. (4) GIPS-III: the title of this trial is Metabolic Modulation with Metformin to Reduce Heart Failure After Acute Myocardial Infarction: Glycometabolic Intervention as Adjunct to Primary Coronary Intervention in ST Elevation Myocardial Infarction (GIPS-III): a Randomized Controlled Trial. The intervention is Metformin vs Placebo. (5) RELAX: the title of this trial is Phosphodiesterase-5 Inhibition to Improve Clinical Status and Exercise Capacity in Diastolic Heart Failure (RELAX). The intervention is Sildenafil vs Placebo. After evaluating the five selected trials, we further chose the WARCEF trial as the final selection based on its ranking in complexity and feasibility.

Table 2 provides a detailed comparison between the traditional randomized clinical trial (RCT) design and our target trial emulation approach. It outlines key aspects, including study aim, eligibility criteria, treatment strategies, follow-up period, and outcome assessment. This comparison highlights the methodological differences, particularly in how the target trial emulation leverages real-world data (RWD) to approximate an RCT while addressing feasibility and generalizability in clinical research.

### 3.2. Statistical Results

We examine the selected WARCEF trial and classify the criteria into three categories: Essential Criteria, Confounding Variable Criteria, and Non-Essential Criteria. For example, in the WARCEF trial, the intervention/treatment criteria, such as aspirin (325 mg per day), are classified as essential criteria since they directly define the treatment being studied. Similarly, the warfarin dosage (INR 2.5–3.0; target INR 2.75) is considered a confounding variable because it influences both the treatment assignment and the outcome, requiring appropriate statistical adjustments. Meanwhile, criteria such as a patient’s ability to adhere to an outpatient protocol, including monthly blood tests, clinic visits every four months, and telephone availability, are categorized as non-essential criteria, as they primarily reflect logistical considerations rather than fundamental study variables.

**Figure 2.**
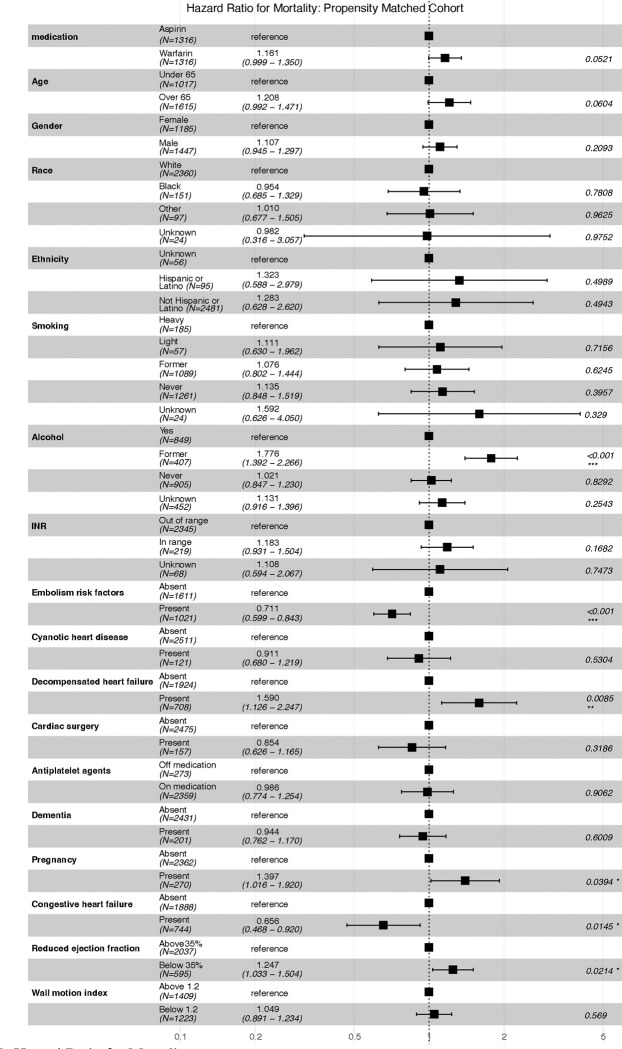
Hazard Ratio for Mortality.

Figure 2 presents a hazard ratio analysis for mortality in a propensity-matched cohort, illustrating the impact of various factors on survival. The comparison between Aspirin and Warfarin suggests that Warfarin may be associated with a slightly higher mortality risk, though the result is not statistically significant. Age and gender do not appear to have a strong effect, though individuals over 65 show a trend toward increased mortality. Lifestyle factors, such as former alcohol use, significantly correlate with higher mortality, while smoking does not show a clear pattern. Among clinical conditions, decompensated heart failure and reduced ejection fraction are linked to higher mortality risk, whereas the presence of embolism risk factors appears to have a protective effect. Pregnancy also shows a significant association with increased mortality. The model, while statistically significant, has moderate predictive accuracy, indicating that while certain factors strongly influence survival, others contribute less definitively.

**Figure 3.**
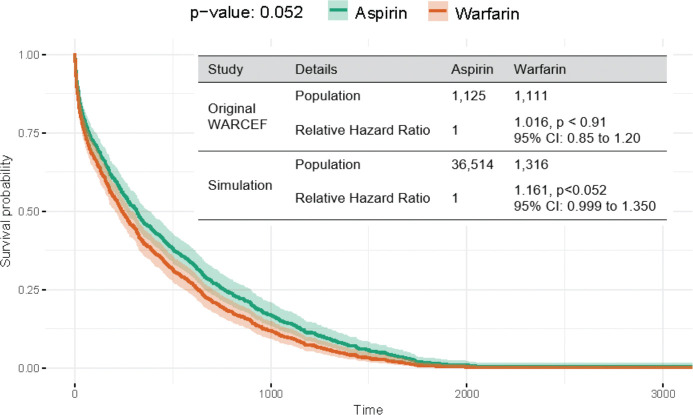
Survival Probability vs Time.

Figure 3 illustrates the Kaplan-Meier survival curves for patients on Aspirin (green) and Warfarin (orange) over time. The y-axis represents survival probability, while the x-axis represents time in days. The shaded areas around each curve indicate the confidence intervals, showing the range of uncertainty in survival estimates. The plot shows a gradual decline in survival probability over time, with both groups experiencing a reduction in survival. However, the decline is consistently steeper for Warfarin compared to Aspirin, suggesting that patients on Aspirin tend to have better survival outcomes over time. The p-value (0.052) indicates a statistically significant difference between the two survival curves, meaning the observed difference is unlikely due to random chance. Overall, this analysis suggests that Aspirin is associated with a higher survival probability compared to Warfarin, reinforcing its potential benefit as a preferred treatment in this context. We incorporate additional confounders, including age, gender, race, ethnicity, smoking status, alcohol consumption, drug consumption, education, and physical activity, into the Cox model to obtain the final metrics as shown in the Table of Figure 3. We then compare our results with the official trial results published on ClinicalTrials.gov. Since the findings are similar, we conclude that our method successfully emulated the trial.

## 4. Discussion and Conclusion

This study demonstrates the feasibility of emulating randomized controlled trials (RCTs) using real-world data (RWD) from the Mayo Clinic Platform (MCP), providing insights into the comparative effectiveness of Warfarin versus Aspirin in patients with reduced cardiac ejection fraction. By leveraging electronic health records (EHRs) and robust statistical methodologies, we sought to bridge the gap between traditional RCTs and real-world evidence (RWE). The results highlight the potential for high-quality observational analyses to produce findings that closely align with interventional clinical trials, reinforcing the value of RWD in clinical decision-making.

One of the most significant challenges in observational research is ensuring a balance between feasibility and scientific rigor when designing the emulation protocol. Unlike RCTs, treatment assignment is not randomized, making bias and confounding major concerns. To address these issues, we applied propensity score matching (PSM) to approximate the treatment assignment process of RCTs. Additionally, data processing and extraction play a crucial role, particularly in handling unstructured clinical notes using NLP techniques to extract relevant data elements. Beyond matching methods, we incorporated key confounders, managed missing data, and carefully defined time zero and follow-ups to ensure coherence in treatment comparisons. These steps helped reduce bias and enhance the comparability of treatment groups. Our findings suggest that, with a well-structured emulation protocol and robust statistical adjustments, observational studies can yield clinically meaningful insights, serving as a valuable complement to traditional RCT findings.

Despite these strengths, several methodological limitations must be acknowledged. Missing data, unmeasured confounders, and selection biases remain inherent challenges in observational research. Although multiple imputation techniques and sensitivity analyses were used to address these issues, residual confounding cannot be entirely ruled out. Furthermore, while EHR-derived outcomes offer valuable insights, differences in documentation, coding, and clinical practices across healthcare settings may introduce variability in the data. The generalizability of our findings should also be interpreted with caution, as observational cohorts may not fully capture the diversity and strict eligibility criteria of traditional RCT populations.

A key finding of this study is the importance of balancing statistical rigor with clinical relevance when emulating RCTs. While applying stricter eligibility criteria ensures methodological validity, it also reduces sample size and statistical power. Our results suggest that many observational studies may not need to enforce all RCT participation criteria to derive meaningful conclusions. Instead, selecting the most essential criteria while adjusting for confounding variables may provide a pragmatic yet robust approach to RCT emulation.

Looking ahead, advancements in machine learning, artificial intelligence, and high-throughput RCT emulation could further enhance the scalability and precision of observational research. Automated patient matching, real-time data integration, and predictive modeling have the potential to refine cohort selection and improve causal inference in future RCT emulations. Additionally, ongoing validation efforts—such as comparing observational findings to completed and ongoing RCTs—will be crucial in solidifying the role of RWD in regulatory decision-making and personalized medicine. Future research should focus on refining these approaches, validating findings against gold-standard RCTs, and exploring new avenues for efficient, scalable, and cost-effective clinical trial alternatives. By demonstrating the potential of RCT emulation, this study contributes to the growing body of evidence supporting real-world data as a critical tool in modern clinical research, helping bridge the gap between traditional RCT methodologies and real-world clinical practice.

## Supplementary Material

Supplement 1

## Figures and Tables

**Table 1.** Study Summaries and Rankings.

RCT	RCT Number	Rank	MCC Approximate Cohort Size (# Patient size)	Original Study Data

WARCEF	NCT00041938	1	697 (Aspirin) | 137 (Warfarin)	1,125 / 1,111
RAID	NCT01215253	2	135(Ranolazine)| 1765 (Placebo)	510 / 502
HEPEF	NCT01726049	3	207(Sildenafil)|1614(Placebo)	26 / 26
GIPS-III	NCT01217307	4	191 (Metformin) | 736 (Placebo)	191 / 188
RELAX	NCT00763867	5	316 (Sildenafil) | 1107 (Placebo)	98 / 101

**Table 2.** Comparison between Randomized Clinical Trial and Our Target Trial Design.

Trials	Randomized clinical trial (NCT00041938)	Our target trial design

Aim	Warfarin Versus Aspirin in Reduced Cardiac Ejection Fraction (WARCEF) Trial (WARCEF)	Determine whether Warfarin or Aspirin is better for preventing death or stroke in patients with poor heart function

Eligibility criteria	**Inclusion criteria:** Cardiac EF <=35% by radionuclide ventriculography, left ventriculography or quantitive echocardiographic measurement or an echocardiographic Wall Motion Index of <=1.2, within three months of enrollment. The patient’s clinical cardiac state at enrollment should be similar to their state at the time of the qualifying echocardiogram. The qualifying left ventricular function measurement must be obtained at least three months after an MI, coronary bypass grafting, PTCA, and at least one month after pacemaker insertion. Patients scheduled for mitral valve repair should have qualifying echo after surgery.	**Criteria used to build cohort:** Patients with Cardiac EF <=35% (<=35% is used as a covariate in analysis) by radionuclide ventriculography, left ventriculography or quantitive echocardiographic measurement or an echocardiographic Wall Motion Index of <=1.2(<=1.2 is used as a covariate in analysis), within three months of enrollment. The qualifying left ventricular function measurement must be obtained at least three months after an MI, coronary bypass grafting, PTCA, and at least one month after pacemaker insertion.
	Modified Rankin score <=4.	Modified Rankin score <=4 or Rankin score <=4
	Patient must be taking ACE inhibitors. If intolerant of ACE inhibitor, patient must be on angiotensin II receptor blockers or hydralazine and nitrates.	Patients must be taking ACE inhibitors. If intolerant of ACE inhibitor, patient must be on angiotensin II receptor blockers or hydralazine and nitrates.
	Person under 18 years of age.	Patients under 18 years of age.
	**Exclusion criteria:** The presence of any of the following unequivocal cardiac sources of embolism: chronic or paroxysmal AF, mechanical valve, endocarditis, intracardiac mobile or pedunculated thrombus, and valvular vegetation.	**Criteria used as confounder:** The presence of any of the following unequivocal cardiac sources of embolism: chronic or paroxysmal AF, mechanical valve, endocarditis, intracardiac mobile or pedunculated thrombus, and valvular vegetation.

Eligibility criteria	Cyanotic congenital heart disease, Eisenmenger’s syndrome. Decompensated heart failure. Patient needs continuing therapy with intravenous heparin or low molecular weight heparin or a specific antiplatelet agent. Dementia or psychiatric or physical problem that prevents the patient from following an outpatient program reliably. Comorbid conditions that may limit survival to less than five years.	Cyanotic congenital heart disease, Eisenmenger’s syndrome. Decompensated heart failure. Patient needs continuing therapy with intravenous heparin or low molecular weight heparin or a specific antiplatelet agent. Dementia or psychiatric or physical problem that prevents the patient from following an outpatient program reliably. Comorbid conditions that may limit survival to less than five years.
	Pregnancy, or female of childbearing potential who is not sterilized or is not using a medically accepted form of contraception* (see procedure manual). *A pregnancy test is required for all women of childbearing age.	Pregnancy, or female of childbearing potential who is not sterilized or is not using a medically accepted form of contraception* (see procedure manual). *A pregnancy test is required for all women of childbearing age.
	Hospitalization for new diagnosis of onset CHF within the past one month or carotid endarterectomy or pacemaker insertion within the past one month prior to randomization.	Hospitalization for new diagnosis of onset CHF within the past one month or carotid endarterectomy or pacemaker insertion within the past one month prior to randomization.

Treatment strategies	Treatment 1: Aspirin (325 mg per day). Treatment 2: Warfarin [International Normalized Ration (INR) 2.5–3.0; target INR = 2.75].	Cohorts selected with medication administered: Aspirin (Dose is used as a covariate in analysis). Warfarin (INR is used as a covariate in analysis).

Treatment assignment	Randomized	Propensity score matching and inverse probability of treatment weighting.

Follow-up period	2002.10 -- 2011.08	Time Zero: First occurrence of all eligibility criteria satisfied. Follow-up: Up to 6 years from Time Zero

Outcome	Composite Endpoint of Ischemic Stroke, Intracerebral Hemorrhage, or Death.	Endpoint of Death.

## Data Availability

This study involves analysis of de-identified Electronic Health Record (EHR) data via Mayo Clinic Platform Discover. Data shown and reported in this manuscript has been extracted from the EHR using an established protocol for data extraction, aimed at preserving patient privacy. The data has been determined to be de-identified pursuant to an expert’s evaluation, in accordance with the HIPAA Privacy Rule. Any data beyond what is reported in the manuscript, including but not limited to the raw EHR data, cannot be shared or released due to the parameters of the expert determination to maintain the data de-identification. Contact corresponding authors for additional details regarding Mayo Clinic Platform Discover.
