# Supplementary material for: From ‘Negative’ Trial to Positive Clinical Impact: Emulating WARCEF While Accounting for Selection Bias in Trial Timing
Source: medRxiv. 2025 Aug 18:2025.03.19.25324271. Preprint. [Version 3] doi: 10.1101/2025.03.19.25324271 (PMC11957179; doi:10.1101/2025.03.19.25324271)
Supplement: 1 [file NIHPP2025.03.19.25324271V3-supplement-1.pdf]

medRxiv preprint doi: https://doi.org/10.1101/2025.03.19.25324271; this version posted August 18, 2025. The copyright holder for this preprint (which was not certified by peer review) is the author/funder, who has granted medRxiv a license to display the preprint in perpetuity.
It is made available under a CC-BY 4.0 International license .

Appendix

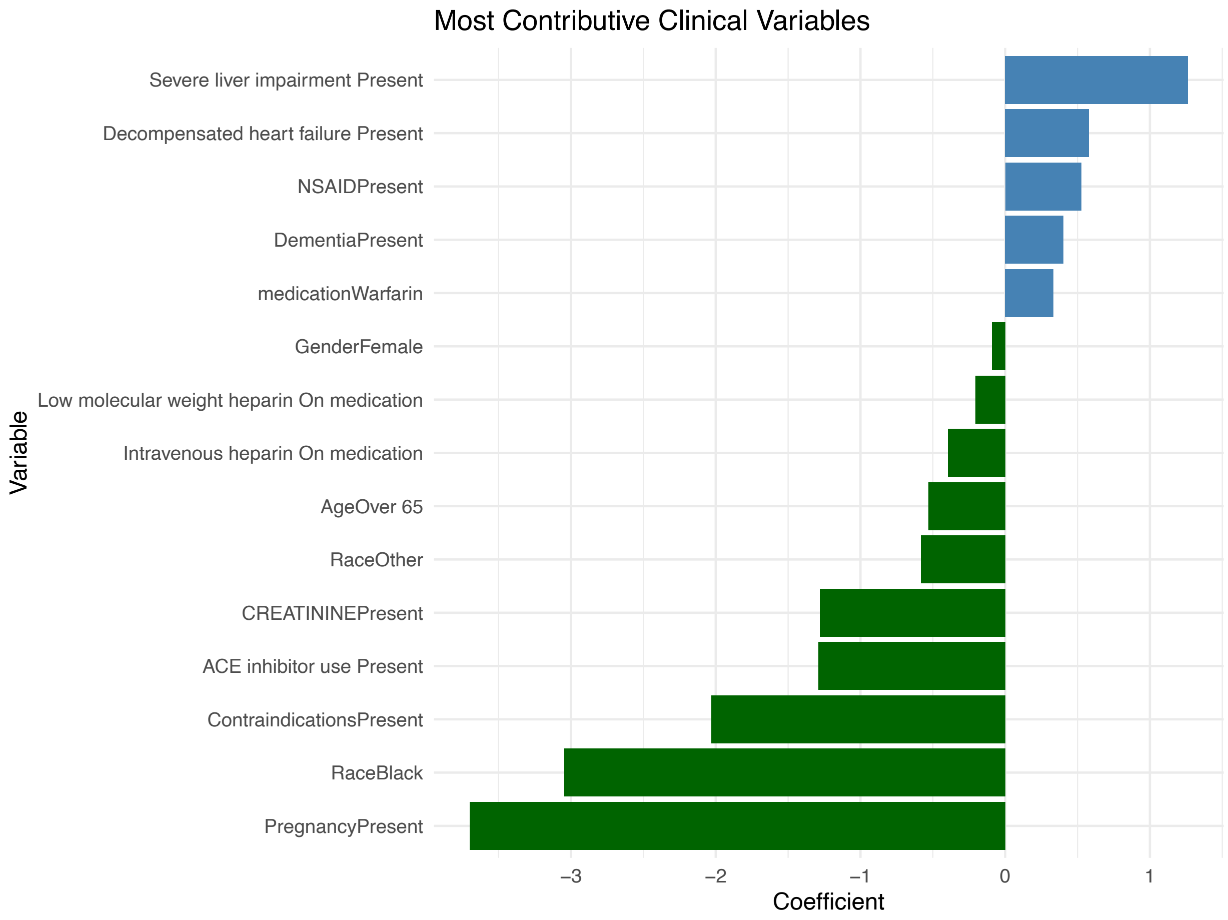

Supplement Figure 1. Confounders Coefficient of ITT before 2014. This figure displays the most influential clinical variables contributing to the outcome in the ITT analysis conducted prior to 2014. The horizontal bar plot ranks variables by the magnitude of their regression coefficients, with variable names listed on the y-axis and their corresponding coefficients on the x-axis. Positive coefficients indicate a stronger association with increased risk or likelihood of the outcome, whereas negative coefficients reflect a protective effect or decreased risk. Among the top positive contributors are variables such as "Severe liver impairment Present" and "Decompensated heart failure Present", suggesting these factors are associated with elevated outcome risk. Similarly, "NSAIDPresent" appear high on the list, reinforcing their clinical importance in the prediction model. On the other hand, several variables demonstrate negative coefficients, indicating a potential protective association. Notably, "PregnancyPresent", "RaceBlack", and "ContraindicationsPresent" have the most negative coefficients, suggesting these factors are linked to reduced risk or lower predicted outcomes in this specific cohort and timeframe. Other negatively associated features include "ACE inhibitor use Present" and "CREATININEPresent", which may reflect their influence in moderating outcome severity. The transition from negative to positive coefficients from top to bottom in the plot highlights the shift in influence among clinical variables. This visualization provides important insight into which factors most strongly affect patient outcomes before 2014, with variables related to liver function, age, and comorbid conditions playing a prominent role in risk stratification.

medRxiv preprint doi: https://doi.org/10.1101/2025.03.19.25324271; this version posted August 18, 2025. The copyright holder for this preprint (which was not certified by peer review) is the author/funder, who has granted medRxiv a license to display the preprint in perpetuity.
It is made available under a CC-BY 4.0 International license .

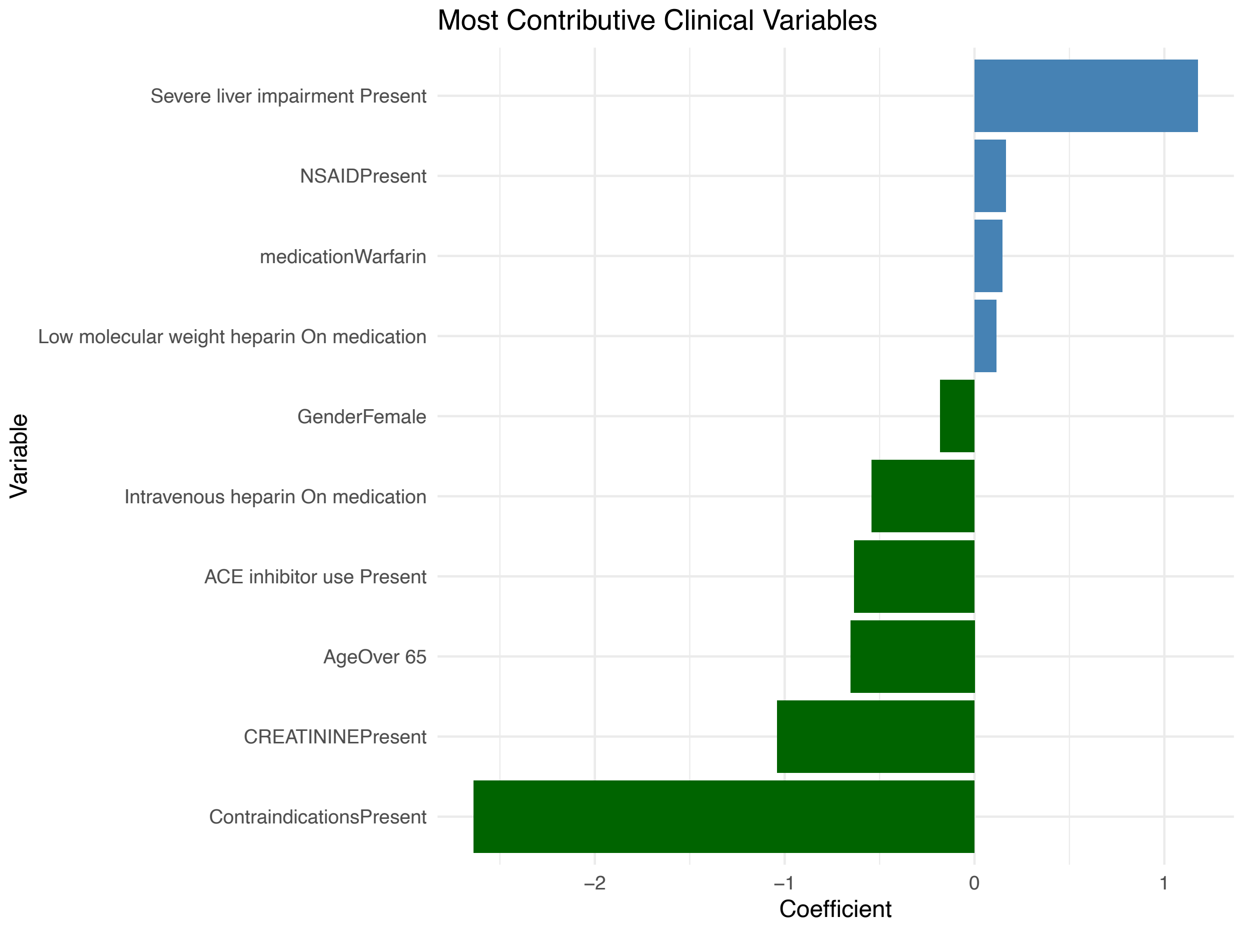

Supplement Figure 2. Confounders Coefficient of PP before 2014. This figure presents the most contributive clinical variables associated with outcomes in the PP analysis prior to 2014. The horizontal bar chart displays clinical variables on the y-axis and their corresponding coefficient values on the x-axis. Positive coefficients indicate an increased risk or likelihood of the outcome, while negative coefficients suggest a protective effect or reduced risk. Among the top positive contributor is "Severe liver impairment Present", is known to be clinically relevant indicators of higher patient risk. Other notable variables with positive associations include "NSAIDPresent", "medicationWarfarin", and "Low molecular weight heparin On medication", indicating that medication use and demographic factors may also influence outcomes in this population. Conversely, several variables demonstrate negative coefficients, pointing to a potential protective effect. Most prominently, "ContraindicationsPresent" has the most negative coefficient, followed by smaller negative contributions from "ACE inhibitor use Present", "Intravenous heparin On medication", and "GenderFemale". These findings may reflect treatment selection patterns or confounding by indication, where certain interventions are linked with lower observed outcome risk under the per-protocol setting. This visualization offers insights into the clinical factors most associated with outcome variation before 2014, with organ dysfunction, age, and contraindications emerging as influential variables within the PP analytic framework.

medRxiv preprint doi: https://doi.org/10.1101/2025.03.19.25324271; this version posted August 18, 2025. The copyright holder for this preprint (which was not certified by peer review) is the author/funder, who has granted medRxiv a license to display the preprint in perpetuity.
It is made available under a CC-BY 4.0 International license .

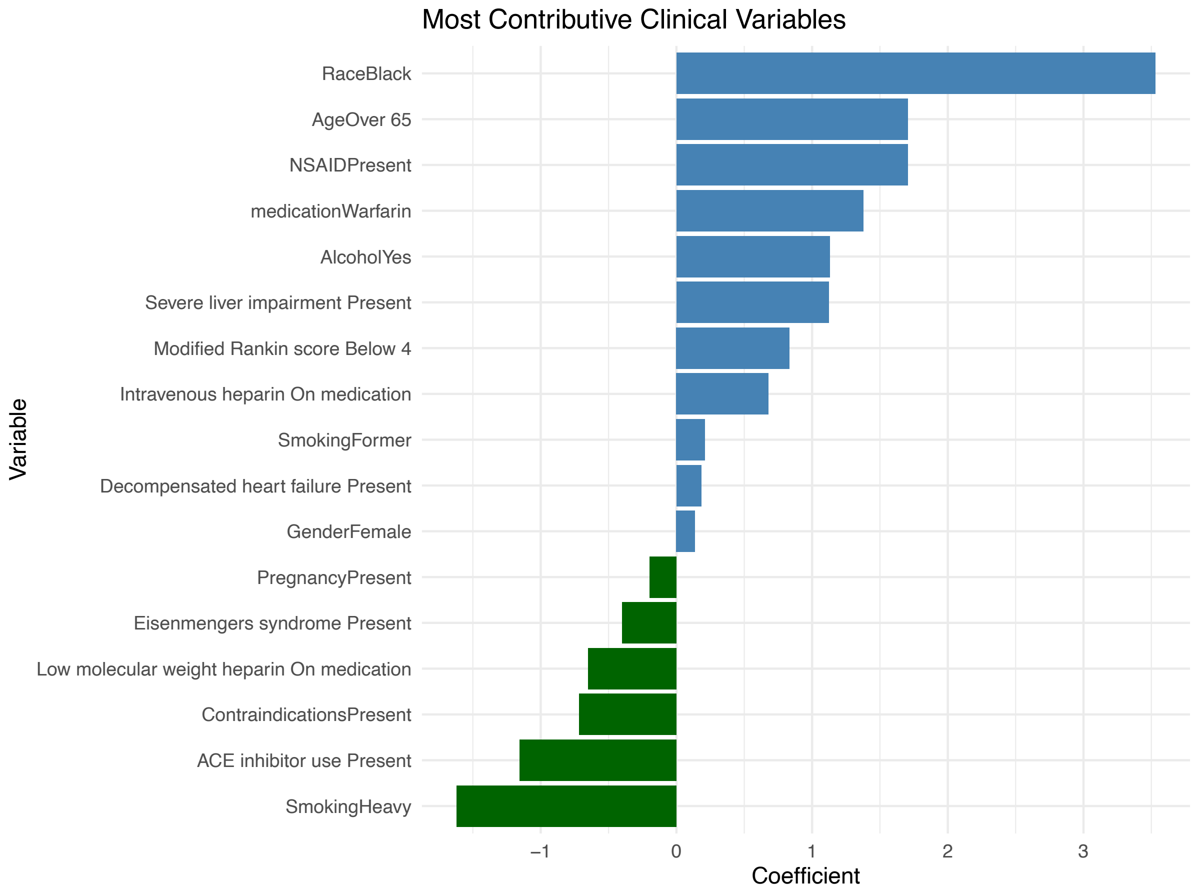

Supplement Figure 3. Confounders Coefficient of PP after 2014. This figure presents the most contributive clinical variables affecting the outcome in the PP analysis conducted after 2014. The horizontal bar plot ranks variables by the magnitude of their coefficients, with variable names listed on the y-axis and corresponding coefficient values on the x-axis. Positive coefficients indicate a stronger association with increased risk or higher probability of the outcome, while negative coefficients reflect a potential protective effect or reduced risk. The most significant positive contributors include "RaceBlack", "AgeOver 65", and "NSAIDPresent", all of which exhibit high positive coefficients, suggesting a strong influence on adverse outcomes. Other notable features with positive associations are "medicationWarfarin" and "AlcoholYes", reinforcing the impact of lifestyle and medication-related variables in this post-2014 cohort. Mid-level contributors include "Severe liver impairment Present", and "Intravenous heparin On medication", which continue to show positive associations with the outcome, albeit to a lesser extent. At the lower end of the coefficient scale, variables such as "Eisenmengers syndromePresent" and "PregnancyPresent" exhibit negative coefficients, indicating a potential protective or less harmful influence on outcomes. "GenderFemale" also appears at the bottom of the list with the smallest (most positive) coefficient. This visualization offers insight into the evolving impact of clinical variables on outcome prediction after 2014, highlighting shifts in demographic and medication-related risk factors, with particular emphasis on age, race, and treatment characteristics.
