# Supplementary material for: Emulating Clinical Trials with the Mayo Clinic Platform: Cardiovascular Research Perspective
Source: medRxiv. 2025 Mar 24:2025.03.19.25324271. Preprint. [Version 2] doi: 10.1101/2025.03.19.25324271 (PMC11957179; doi:10.1101/2025.03.19.25324271)
Supplement: Supplementary file 1 [file NIHPP2025.03.19.25324271v2-supplement-1.pdf]

Appendix

Supplement Table 1. Selected Trials.

NCT Number	Study Title
NCT00041938	Warfarin Versus Aspirin in Reduced Cardiac Ejection Fraction (WARCEF) Trial
NCT01215253	Ranolazine Implantable Cardioverter-Defibrillator Trial
NCT01726049	Sildenafil in HFpEF (Heart Failure with Preserved Ejection Fraction) and PH
NCT01217307	Metformin to Reduce Heart Failure After Myocardial Infarction
NCT00763867	Evaluating the Effectiveness of Sildenafil at Improving Health Outcomes and Exercise Ability in People with Diastolic Heart Failure (The RELAX Study)

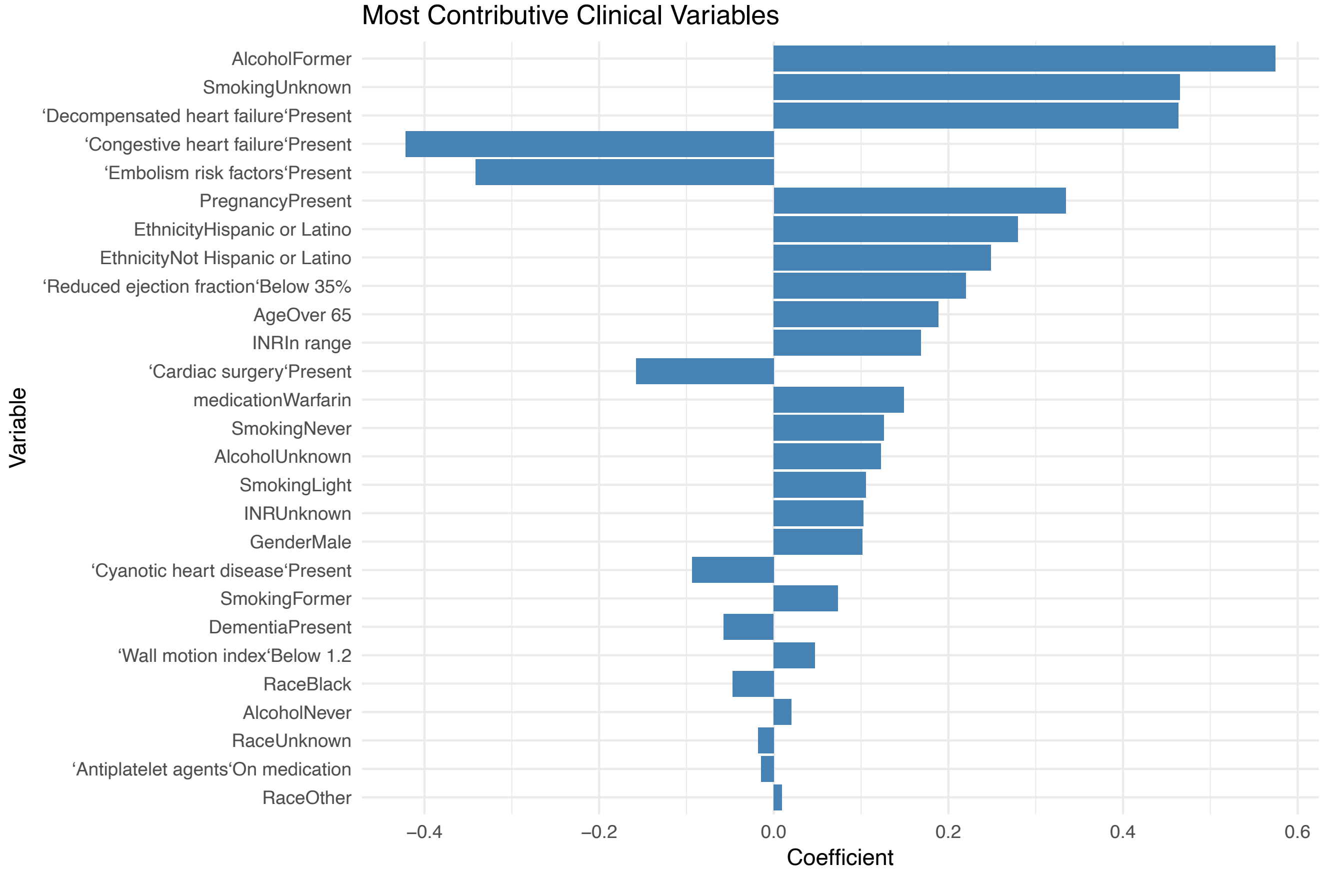

Supplement Figure 1. Confounders Coefficient. This figure presents the most contributed clinical variables affecting the outcome, ranked by their coefficient values. The horizontal bar plot displays variable names on the y-axis and their corresponding coefficients on the x-axis, with positive values indicating a stronger association with increased risk or outcome probability and negative values representing protective or lower-risk factors. The top contributors include "AlcoholFormer" and "SmokingUnknown", which have the highest positive coefficients, suggesting a strong influence on the outcome. Similarly, "Decompensated heart failure" and "Congestive heart failure" (both marked as present) also show large positive contributions, indicating a strong association with worsening outcomes. "Embolism risk factors" and "Pregnancy" follow closely, reinforcing their clinical relevance. Conversely, variables with negative coefficients (lower down the chart) suggest protective effects or reduced risk. For instance, "RaceOther", "Antiplatelet agents on medication", "RaceUnknown", and

"AlcoholNever" have the lowest coefficients, indicating a weaker or negative association with the outcome. Additionally, variables such as "Cyanotic heart disease present," "Dementia present," and "Wall motion index below 1.2" appear near the lower end, suggesting a lesser impact compared to the top contributors. The transition from high to low coefficients, as indicated from top to bottom, highlights the decreasing influence of variables on the outcome. This visualization provides valuable insights into which clinical factors play a dominant role in predicting patient outcomes, with conditions like heart failure, smoking, and embolism risk factors being the most significant contributors.
